# Risk of Second Primary Malignancies among Patients with Early Gastric Cancer Exposed to Recurrent Computed Tomography Scans

**DOI:** 10.3390/cancers13051144

**Published:** 2021-03-07

**Authors:** Tae Jun Kim, Yeong Chan Lee, Yang Won Min, Hyuk Lee, Byung-Hoon Min, Jun Haeng Lee, Hong-Hee Won, Kyoung Doo Song, Woo Kyoung Jeong, Jae J. Kim

**Affiliations:** 1 Department of Medicine, Samsung Medical Center, Sungkyunkwan University School of Medicine, Seoul 06351, Korea; taejunk91@skku.edu (T.J.K.); yangwon.min@samsung.com (Y.W.M.); lhyuk.lee@samsung.com (H.L.); jason.min@samsung.com (B.-H.M.); jh2145.lee@samsung.com (J.H.L.); 2 Department of Digital Health, SAIHST, Sungkyunkwan University School of Medicine, Seoul 06351, Korea; conan_8th@naver.com (Y.C.L.); honghee.won@samsung.com (H.-H.W.); 3 Department of Radiology, Samsung Medical Center, Sungkyunkwan University School of Medicine, Seoul 06351, Korea; kd3893.song@samsung.com (K.D.S.); wookyoung.jeong@samsung.com (W.K.J.)

**Keywords:** computed tomography, early gastric cancer, radiation exposure

## Abstract

**Simple Summary:**

Cancer risk after radiation exposure during childhood has been extensively documented in the literature, although cancer risk associated with recurrent computed tomography (CT) scans during adulthood is less understood. We found a significant relationship between the frequency of CT scans and the subsequent incidence of secondary primary malignancies in patients who have undergone curative resection for early gastric cancer (EGC). On the basis of the low incidence of extragastric recurrence and the risk of radiation exposure, we suggest that overzealous CT surveillance should be avoided in adult patients with EGC.

**Abstract:**

Although computed tomography (CT) scans are very useful for identification or surveillance of malignancy, they are also associated with the risk of cancer caused by ionizing radiation. We investigated the risk of second primary malignancies (SPMs) after frequent abdominopelvic CT scans in a cohort of Korean patients with early gastric cancer (EGC). We performed a cohort study of 11,072 patients who underwent resection for EGC at Samsung Medical Center and validated the results using data from 7908 patients in a Korean National Health Insurance Service cohort. Cox proportional hazards regression model was used to estimate hazard ratios (HRs) for intra-abdominal SPM. During 43,766.5 person-years of the follow-up at our center, 322 patients developed intra-abdominal SPMs. Patients who underwent receiving >8 abdominopelvic CT scans had a significantly greater risk of developing SPM (HR, 2.73; 95% CI, 1.66–4.50; *p* < 0.001) than those who had with ≤8 scans. For each additional abdominopelvic CT scan, the adjusted HR for SPM was 1.09 (95% confidence interval (CI), 1.03–1.14). Similar results were observed in the Korean National Health Insurance Service cohort (adjusted HR, 1.14; 95% CI, 1.07–1.22). Significantly elevated risk of SPM was still observed when considering a 2-year latency period (adjusted HR, 2.43; 95% CI, 1.37–4.48) and a 3-year latency period (adjusted HR, 2.17; 95% CI, 1.06–4.47). Frequent abdominopelvic CT scans are associated with an elevated risk of SPMs after the treatment of EGC. Thus, physicians need to weigh carefully the clinical benefits of CT examinations against the potential risks of radiation exposure.

## 1. Introduction

Early gastric cancer (EGC) is defined as gastric cancer that is confined in the mucosal or submucosal layer, regardless of lymph node metastasis. In Korea and Japan, gastric cancer is more often detected in the early stages because of national cancer screening programs and regular health check-ups [1,2]. Gastrectomy with lymph node dissection has been the standard treatment for EGC, and endoscopic resection is accepted as the standard treatment for EGC with a negligible risk of lymph node metastasis [3,4,5]. Surgical or endoscopic resection for EGC achieves excellent long-term outcomes with a 10-year overall survival rate of more than 94% [4]. Intragastric recurrence is detected with scheduled endoscopic surveillance, and extragastric recurrence is mostly diagnosed using abdominopelvic computed tomography (CT) scans. Recently, there have been several reports regarding the unclear role of CT in surveillance for EGC due to the low incidence rate of extragastric recurrence after the treatment of EGC [6,7,8,9]. 

The use of CT has been increased rapidly based on its value in most fields of medicine, especially in the screening or surveillance of malignancies. The development of high-speed multidetector CT scanners has facilitated the capture of images with greater definition during shorter scans, which has also supported the increased use of CT. The rates of CT use have been increasing rapidly in the United States and in other regions [10], and Korea has seen an increase of approximately 20% each year, from 1.7 million scans in 2003 to 4.8 million scans in 2009. The increased use of CT has also attracted interest in the risks of radiation-induced cancer that are associated with frequent CT scans [11,12]. Brenner et al. reported that 1.5–2.0% of all cancers in the United States may be attributable to CT-related radiation [11]. 

To the best of our knowledge, there has been no study on the risk of secondary primary malignancies (SPMs) due to recurrent radiation exposure in patients with resected EGC. Recent studies reported that CT had an unclear role in the surveillance after the treatment of EGC; however, they did not describe the potential risk of CT radiation in detail [6,7,8,9,13]. Therefore, we aimed to evaluate whether the risk of SPM was elevated among patients with EGC exposed to recurrent abdominopelvic CT scans.

## 2. Materials and Methods

### 2.1. Study Population

**Figure 1:**
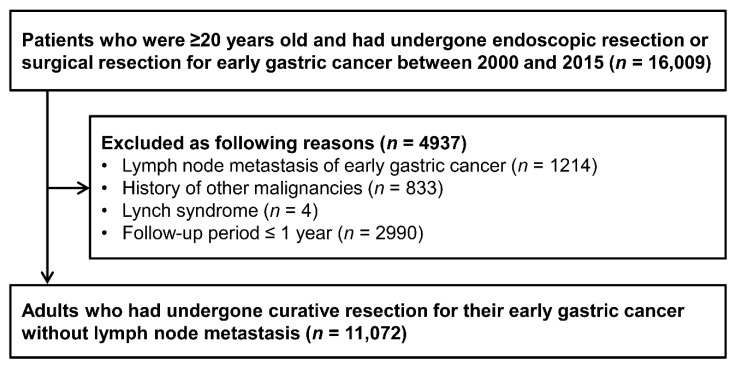
Flowchart of patient selection criteria.

We performed a hospital-based retrospective cohort study of patients who underwent endoscopic resection or gastrectomy for EGC at Samsung Medical Center (Seoul, Korea). Patients were considered eligible if they were ≥20 years old and underwent endoscopic or surgical resection for EGC without lymph node metastasis between 1 January 2000, and 31 December 2018 (n = 16,009). However, we excluded the following patients: those who had lymph node metastasis of EGC (n = 1214), who had other malignancies within 5 years before index date (n = 833), who did not have a minimum follow-up of 3 years (n = 2990), or who had Lynch syndrome (n = 4). Thus, we ultimately included 11,072 adults from the initial cohort of patients who underwent endoscopic or surgical resection for their EGC, did not have a history of other malignancy, and were followed up for >3 years (Figure 1). 

We have also evaluated data from a validation cohort that was derived from the National Health Insurance Service (NHIS) database between 1 January 2005 and 31 December 2017. Korea has a single-payer national health insurance system (NHIS) that covers almost the entire Korean population and maintains records regarding all reimbursements for inpatient visits, outpatient visits, procedures, and prescriptions. We also searched the NHIS database for patients who were aged ≥20 years and had undergone endoscopic resection for EGC (n = 9374). We excluded patients with a history of malignancy, those who underwent gastrectomy before or after endoscopic resection, and patients without a minimum follow-up of 3 years (n = 1466). Thus, the NHIS cohort ultimately included 7908 patients (5606 men and 2302 women). The study protocol was reviewed and approved by our institutional review board, which waived the requirement for obtaining informed patient consent, as deidentified data were used in this study.

### 2.2. Study Outcomes

The primary outcome was defined as the development of intra-abdominal non-gastric solid cancers during the follow-up period. These cases were identified based on the International Classification of Disease, 10th revision (ICD-10) codes C00–C96, although we did not consider cases with a second gastric cancer (ICD-10: C16) in the analysis. 

Exposure was defined as the number of abdominopelvic CT scans (a continuous variable) from 5 years before to 3 years after the index date. The effects of radiation exposure from CT scans were also evaluated by dividing the patients into two groups (dichotomized) and three groups (tertiles). The index date was defined as the date of the primary EGC resection, and patients were followed up from the index date to the first instance of a secondary cancer diagnosis, death, the last clinic visit, or 31 January 2020, whichever occurred first. We excluded patients who were followed up for <3 years or who developed cancer within 3 years after the index date. The main analyses were based on a latency period of 1 year, although we also explored the possibility of reverse causation based on latency periods of 2 and 3 years. 

### 2.3. Covariates

At the index date, we collected data on patient age, sex, smoking status, and comorbidities. Smoking status was categorized as never, former, or current smoker. Comorbidities during the 5-year period before the index date were identified using ICD-10 codes and summarized using the Charlson Comorbidity Index (CCI). The CCI included myocardial infarction, congestive heart failure, peripheral vascular disease, cerebrovascular disease, dementia, chronic pulmonary disease, connective tissue disease, peptic ulcer disease, diabetes without complications, diabetes with complications, paraplegia/hemiplegia, mild liver disease, moderate or severe liver disease, and renal disease. The ICD-10 codes for these comorbidities are described in Table S1. 

### 2.4. Statistical Analysis

Continuous variables were reported as mean (standard deviation), and categorical variables were reported as number (percentage). Continuous variables were compared using Student’s *t*-test, and categorical variables were compared using the chi-square test. Descriptive statistics were used to summarize patients’ baseline characteristics according to sex. The primary endpoint was the development of secondary cancer among the study’s patients with resection for EGC. The last follow-up date was defined as the earliest date among the date of diagnosis of secondary cancer, death, or the last hospital visit (31 January 2020). To avoid the inclusion of CT scans related to cancer diagnosis, we performed the main analyses with a latent period of 1 year and, in addition, we performed sensitivity analyses with latent periods of 2 and 3 years. The incidence of cancer was calculated as the number of cancer cases per 1000 person-years. We also used a Cox proportional hazard model to estimate the adjusted hazard ratio (HR) and a 95% confidence interval (CI) for developing CT scan-associated cancer, which was adjusted for patient age (each additional year), sex (male vs. female), smoking status (never vs. former or current smoker), and comorbidities (based on the CCI). The cumulative incidences of intra-abdominal cancer were evaluated using Kaplan–Meier curves and the log-rank test. 

Subgroup analyses were also performed according to patient age (<50 years vs. ≥50 years), sex (female vs. male), smoking status (never vs. former or current smoker), CCI score (<2 vs. ≥2), and chronic hepatitis or liver cirrhosis (no vs. yes). Differences were considered statistically significant at *p*-values of <0.05, and all analyses were performed using SAS software (version 9.4; SAS Institute, Cary, NC, USA) and R software (version 3.5.1; R Foundation, Vienna, Austria). 

## 3. Results 

Table 1 shows the demographic and clinical characteristics of the 11,072 adult patients who underwent endoscopic or surgical resection for EGC between 2000 and 2015, had no history of other malignancy, and were followed up for >3 years. We collected data regarding 32,653 abdominopelvic CT scans that were performed 5 years before the index date to 3 years after the index date. The indications or reasons for CT are shown in Table S2. Mean patient age was 56.5 years (standard deviation, 11 years); among 11,072 patients, there were 7412 men (66.9%) and 3660 women (33.1%). 

**Figure 2:**
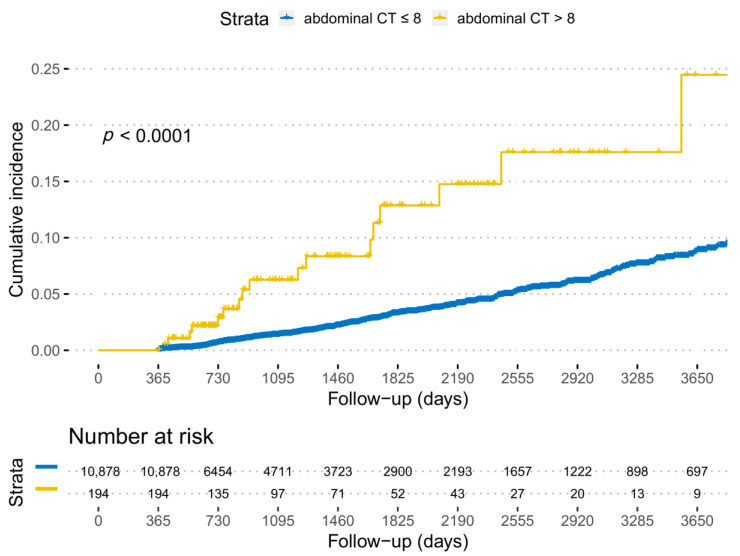
Ten-year cumulative incidence of intra-abdominal second primary malignancies following abdominopelvic computed tomography scans ≤8 versus >8.

During 43,766.5 person-years of follow-up, 322 patients developed intra-abdominal SPMs. The incidence of intra-abdominal SPM in patients who had more abdominopelvic CT scans was significantly higher than that in patients who had fewer CT scans (Figure S1). A receiver-operating characteristic (ROC) curve analysis was performed to evaluate the predictive performance of abdominopelvic CT scans on SPM development. The area under the curve of abdominopelvic CT scans was 0.736. Based on the ROC curve analysis, the cut-off value of the numbers of abdominopelvic CT scans was 9. The multivariable analyses revealed increased risks of intra-abdominal SPM >8 abdominopelvic CT scans (adjusted HR, 2.73; 95% CI, 1.66–4.50) (Table 2). The Kaplan–Meier curve analysis plot is shown in Figure 2. The risks of intra-abdominal SPM were also elevated for the age of ≥50 years (adjusted HR, 2.64; 95% CI, 1.87–3.73), male sex (adjusted HR, 1.61; 95% CI, 1.17–2.21), and former or current smokers (adjusted HR, 1.58; 95% CI, 1.23–2.03). The risk of SPMs was significantly increased with additional abdominopelvic CT scans (adjusted HR, 1.09; 95% CI, 1.03–1.14) (Table 3). Among the SPM, additional abdominopelvic CT scans were associated with increased risks of liver, pancreatic, renal, and bladder cancers. In the nationwide cohort, additional abdominal CT scans were associated with an increased risk of intra-abdominal SPM (adjusted HR, 1.14; 95% CI, 1.07–1.22).

To avoid surveillance bias, we also evaluated the risk of intra-abdominal SPMs associated with abdominopelvic CT scans for various latency periods (Table 4). Similar to the results of a 1-year latency period (abdominopelvic CT scans >8 versus ≤8: adjusted HR, 2.48; 95% CI, 1.37–4.48), abdominopelvic CT scans were associated with increased risks using a 2-year latency period (adjusted HR, 2.48; 95% CI, 1.37–4.48) and a 3-year latency period (adjusted HR, 2.17; 95% CI, 1.06–4.47). We also evaluated the CT-related risks of developing intra-abdominal SPMs in various patient subgroups (Table S3). The pre-specified subgroup analysis demonstrated no heterogeneity in the risk of SPM associated with abdominopelvic CT scans, or significant interactions by age (<50 years vs. ≥50 years), sex (male vs. female), smoking status (never vs. past or current smoker), and comorbidities (<2 vs. ≥2). 

## 4. Discussion 

The present study evaluated patient data from a large hospital-based cohort, which revealed a significant dose–response relationship between radiation from abdominopelvic CT scans and the subsequent incidence of intra-abdominal SPM in patients with EGC. We also found that the risk of radiation-related SPMs remained significant at a latency period of up to three years. These results indicate that recurrent abdominopelvic CT scans likely lead to radiation exposure that increases the risk of SPM in patients with EGC. 

The exact risk of cancer from CT continues to be a subject of debate. There is uncertainty about the exact carcinogenic risk due to exposure to low doses of radiation. In addition, different CT types have wide variability in the range of radiation dose. Previous studies reported that the effective dose by body site is 2 mSv for head CT, 8 mSv for chest CT, 10 mSv for abdominal CT, and 10 mSv for CT colonography [14,15]. The best available data regarding the risks of radiation-induced cancer come from Japanese atomic bomb survivors because this cohort has a large population with a long follow-up period [16]. The data reported an increased risk of overall cancer after exposure to low doses of radiation, ranging from 5 to 150 mSv [17,18]. The radiation doses in the range are those that can be reached during recurrent CT scans.

Several epidemiological studies have revealed an increased cancer risk after pediatric CT scans [10,19,20,21,22,23,24]. The first study conducted in the United Kingdom revealed an increased incidence of leukemia and brain tumor after undergoing CT scans in childhood [23]. Furthermore, Australian investigators revealed that people who underwent CT scans before the age of 20 years had an increased incidence of several cancer types, including digestive organ tumors, thyroid tumors, and brain tumors [21]. Moreover, a study on Taiwanese people who underwent at least one CT scan before the age of 18 years revealed an increased incidence of benign brain tumors and that the frequency of the CT scans was strongly correlated with the risk of developing benign brain tumors [19]. A recent study from the Netherlands also revealed an association between radiation exposure during pediatric CT scans and the subsequent risk of developing brain tumors [24]. Fortunately, many radiologists are now aware of the risks and use related methods to reduce the dose at each examination, which include technical developments, imaging parameter selection, protocol modifications, and utilization of standardized reference dose levels [25,26]. Nevertheless, it is important to educate non-radiology physicians, who are responsible for ordering most CT scans, regarding the need to restrict CT utilization to definite clinical indications as well as non-ionizing alternatives when they are feasible. 

Recent population-based studies conducted in the United States have revealed that approximately 50% of the population underwent as least one CT scan during the 10-year study period [27]. Furthermore, the abdomen and pelvis were the most common CT scan sites and accounted for 67.2% of the total radiation dose. Among patients who received high-radiation doses through CT scanning, approximately 50% of patients underwent the procedure for restage cancers or abdominal pain. Furthermore, a recent study indicated that repetitive CT scans were performed for the diagnosis and conservative management of abdominal and pelvic diseases [28]. Among patients who underwent CT scans, 33% had ≥5 CT scans during their lifetime and 5% underwent 22–133 CT scans during their lifetime [29]. This may indicate that many abdominal CT scans are clinically unnecessary, and our results suggest that their benefits do not outweigh the risk of radiation-associated cancer.

To the best of our knowledge, this is the first study to describe a significantly higher risk of second cancer in patients with EGC who had frequent CT scans. Several studies investigated the incidence of extragastric recurrence after the resection of EGC [6,7,8,9,13]. Park et al. reported that no extragastric recurrence developed during a median follow-up of 19.7 months after endoscopic resection for EGC [13]. Other studies showed that the incidence rates of extragastric recurrence after endoscopic resection for early gastric cancer were 0.15% (2/1306), 0.37% (15/4105), and 0.5% (2/404) [6,8,9]. The incidence of extragastric recurrence after surgical resection of EGC ranged from 1.4% to 2.2%, which was slightly higher than that after endoscopic resection [7,30]. Although the incidence of extragastric recurrence is low, CT surveillance is necessary considering significant morbidity and mortality of recurrence. In some institutions, abdominopelvic CT scans are performed every 6 months within 2 or 3 years after the treatment of EGC [6,31,32,33]. This study suggests that overzealous CT surveillance should be avoided in patients with EGC based on the low incidence of extragastric recurrence and cancer risk because of radiation exposure. 

In the present study, the most common sites of radiation-associated cancers were the liver, pancreas, renal, and bladder. Studies of Thorotrast-exposed patients have also provided conclusive evidence that the radiation had a carcinogenic effect on the liver. Reports on Japanese atomic bomb survivors have also revealed a significant dose-related increase in renal cancer cases [34]. According to a report from the United Nations Commission on the Effect of Ionizing Radiation, there is convincing evidence for a relationship between exposure to ionizing radiation and bladder cancer [35]. The present study also revealed that pancreatic cancers were predominant among patients who underwent a larger number of CT scans, which implies that there is an elevated risk at the overlapped anatomical sites. 

The present study has several limitations that warrant consideration. First, we did not have access to information regarding factors that may be related to the development of specific cancer types, environmental exposure to chemicals, history of substance use, or dietary patterns. Therefore, it is possible that these factors had confounding effects on our findings. Second, the small number of cases of rare cancers may have reduced the statistical power of the related analyses. Nevertheless, this study included a relatively large cohort over several years of the follow-up. Third, this study focused on Korean adults who underwent resection for EGC, and our findings may not be generalized to other populations. However, based on the homogeneous patient sample with regular follow-up visits, our results appear to support the dose–response relationship between recurrent CT scans and secondary cancer risk.

## 5. Conclusions

In conclusion, this study demonstrated that overzealous CT use in patients with EGC increases the risk of SPM. Thus, physicians need to weigh carefully the clinical benefits of CT examinations against the potential risks of radiation exposure. On the basis of the low incidence of extragastric recurrence and the risk of radiation exposure, we suggest that overzealous CT surveillance should be avoided in patients with EGC. In this study, there was a significant increase in the risk of cancer from nine or more CT scans. Further studies with a larger population and a longer follow-up period are needed to determine the appropriate risk–benefit balance in recurrent CT scans. 

## Figures and Tables

**Table 1:** Baseline characteristics of patients in the study cohort.

	Total	Male Patients	Female Patients	*p* Value
Number of patients	11,072	7412	3660	
Age (years)	56.5 ± 11.03	57.3 ± 10.4	54.8 ± 11.8	<0.001
<50	2962 (26.8%)	1724 (23.3%)	1238 (33.8%)	
≥50	8110 (73.2%)	5688 (76·7%)	2422 (66.2%)	
Smoking status				<0.001
Never	6875 (62.1%)	3346 (45.1%)	3529 (96.4%)	
Past	2369 (21.4%)	2303 (31.1%)	66 (1.8%)	
Current	1828 (16.5%)	1763 (23.8%)	65 (1.8%)	
Comorbidities				
Myocardial infarction	51 (0.5%)	46 (0.6%)	5 (0.1%)	<0.001
Congestive heart failure	61 (0.6%)	51 (0.7%)	10 (0.3%)	0.008
Peripheral vascular dis.	72 (0.7%)	66 (0.9%)	6 (0.2%)	<0.001
Cerebrovascular dis.	348 (3.1%)	258 (3.5%)	90 (2.5%)	0.004
Dementia	29 (0.3%)	17 (0.2%)	12 (0.3%)	0.449
Chronic pulmonary dis.	362 (3.3%)	285 (3.8%)	77 (2.1%)	<0.001
Connective tissue dis.	32 (0.3%)	9 (0.1%)	23 (0.6%)	<0.001
Peptic ulcer dis.	1375 (12.4%)	996 (13.4%)	379 (10.4%)	<0.001
Diabetes (without complications)	643 (5.8%)	496 (6.7%)	147 (4.0%)	<0.001
Diabetes (with complications)	91 (0.8%)	69 (0.9%)	22 (0.6%)	0.090
Paraplegia/hemiplegia	5 (0.0%)	5 (0.1%)	0 (0.0%)	0.273
Mild liver disease	445 (4.0%)	332 (4.5%)	113 (3.1%)	0.001
Moderate or severe liver disease	1 (0.0%)	1 (0.0%)	0 (0.0%)	1.000
Renal disease	91 (0.8%)	72 (1.0%)	19 (0.5%)	0.018

Values are expressed as means ± standard deviation or numbers (percentages).

**Table 2:** Risk factors associated with intra-abdominal SPM development.

Covariates.	Adjusted HR (95% CI)
Abdomen (with pelvis) CT scans	
Continuous variable	1.09 (1.03–1.14)
Binary	
≤8	Reference
>8	2.73 (1.66–4.50)
Tertile	
0–6	Reference
7–8	1.31 (0.95–1.81)
>8	2.85 (1.72–4.71)
Age (continuous variable)	1.06 (1.05–1.07)
<50	Reference
≥50	2.64 (1.87–3.73)
Sex	
Female	Reference
Male	1.61 (1.17–2.21)
Smoking status	
Never	Reference
Past or current	1.58 (1.23–2.03)
Comorbidities	
Myocardial infarction	0.78 (0.18–3.39)
Congestive heart failure	0.54 (0.07–3.90)
Peripheral vascular dis.	0.14 (0.02–1.01)
Cerebrovascular dis.	0.99 (0.58–1.69)
Dementia	0.95 (0.13–7.02)
Chronic pulmonary dis.	0.91 (0.54–1.54)
Connective tissue dis.	
Peptic ulcer dis.	1.13 (0.81–1.57)
Diabetes (without complications)	1.00 (0.64–1.55)
Diabetes (with complications)	1.12 (0.46–2.68)
Paraplegia/hemiplegia	2.33 (0.28–19.50)
Mild liver disease	1.95 (1.35–2.81)
Moderate or severe liver disease	
Renal disease	0.60 (0.15–2.45)

Estimated from Cox proportional hazard models and the multivariable model included all the above variables; SPM, second primary malignancy; HR, hazard ratio; CI, confidence interval.

**Table 3:** Risk of SPM following abdominopelvic CT scans (continuous variable).

	Cohort (n = 11,072)			NHIS Cohort (n = 7908)		
	No. of Cases	Incidence Rate (per 1000 Person-Years)	Adjusted HR (95% CI)	No. of Cases	Incidence Rate (per 1000 Person-Years)	Adjusted HR (95% CI)
Intra-abdominal SPM	322	7.36	1.09 (1.03–1.14)	271	8.82	1.14 (1.07–1.22)
Liver	50	1.14	1.10 (1.01–1.20)	58	1.89	1.16 (1.01–1.34)
Gallbladder and biliary tract	41	0.94	0.90 (0.63–1.28)	18	0.59	1.24 (0.97–1.58)
Pancreas	33	0.75	1.28 (1.15–1.43)	32	1.04	1.36 (1.18–1.57)
Small bowel	3	0.07	1.37 (0.65–2.92)	0	0	-
Colon and rectum	83	1.9	1.04 (0.92–1.17)	53	1.73	0.87 (0.69–1.10)
Kidney	10	0.23	1.29 (1.03–1.62)	12	0.39	1.14 (0.72–1.82)
Bladder	37	0.85	1.25 (1.11–1.41)	19	0.62	1.17 (0.91–1.49)
Prostate	60	1.37	1.09 (0.99–1.21)	75	2.44	1.13 (0.99–1.29)
Cervix uteri	3	0.07	1.19 (0.58–2.45)	0	0	-
Corpus uteri	1	0.02	1.18 (0.34–4.11)	0	0	-
Ovary	1	0.02	1.46 (0.34–6.19)	4	0.13	1.19 (0.62–2.27)

Estimated from Cox proportional hazard models adjusted for age, sex, smoking status, and Charlson comorbidity index. SPM, second primary malignancy; HR, hazards ratio; CI, confidence interval; CNS, central nervous system.

**Table 4:** Risk of intra-abdominal SPMs following abdominopelvic CT scans according to the latent period.

Latent Period	Adjusted HR (95% CI) Continuous Variable	*p* Value	Adjusted HR (95% CI) 8> Versus ≤8 (Reference)	*p* Value
1 years	1.09 (1.03–1.14)	0.001	2.73 (1.66–4.50)	<0.001
2 years	1.09 (1.03–1.15)	0.005	2.48 (1.37–4.48)	0.003
3 years	1.06 (0.99–1.14)	0.105	2.17 (1.06–4.47)	0.035

Estimated from Cox proportional hazard models adjusted for age, sex, smoking status, and Charlson Comorbidity Index. SPM, second primary malignancy; HR, hazards ratio; CI, confidence interval.

## Data Availability

The data presented in this study are available on request from the corresponding author. The data are not publicly available due to privacy and ethical restrictions.

## Supplementary Materials

The following are available online at https://www.mdpi.com/2072-6694/13/5/1144/s1, Table S1. ICD-10 codes for comorbidities; Table S2. Indications/reasons for CT; Table S3. Risk of intra-abdominal SPMs following abdominopelvic CT scans in selected population subgroups; Figure S1. Absolute risks for SPMs according to the number of CT scans.
