# Supplementary material for: Risk of Second Primary Malignancies among Patients with Early Gastric Cancer Exposed to Recurrent Computed Tomography Scans
Source: Cancers (Basel). 2021 Mar 7;13(5):1144. doi: 10.3390/cancers13051144 (PMC7962111; doi:10.3390/cancers13051144)
Supplement: Supplementary file 1 [file cancers-13-01144-s001.pdf]

cancers

Supplementary Materials

Risk of Second Primary Malignancies among Patients with Early Gastric Cancer Exposed to Recurrent Computed Tomography Scans

Tae Jun Kim, Yeong Chan Lee, Yang Won Min, Hyuk Lee, Byung-Hoon Min, Jun Haeng Lee, Hong-Hee Won, Kyoung Doo Song, Woo Kyoung Jeong and Jae J. Kim

Table S1. ICD-10 codes for comorbidities.

Covariates	ICD-10 code
Myocardial infarction	I21, I22, I252
Congestive heart failure	I43, I50, I099, I110, I130, I132, I255, I420, I425, I426, I427, I428, I429, P290
Peripheral vascular dis.	I70, I71, I731, I738, I739, I771, I790, I792, K551, K558, K559, Z958, Z959
Cerebrovascular dis.	G45, G46, I60, I61, I62, I63, I64, I65, I66, I67, I68, I69, H340
Dementia	F00, F01, F02, F03, G30, F051, G311
Chronic pulmonary dis.	J40, J41, J42, J43, J44, J45, J46, J47, J60, J61, J62, J63, J64, J65, J66, J67, I278, I279, J684, J701, J703
Connective tissue dis.	M05, M32, M33, M34, M06, M315, M351, M353, M360
Peptic ulcer dis.	K25, K26, K27, K28
Diabetes (without complications)	E100, E101, E106, E108, E109, E110, E111, E116, E118, E119, E120, E121, E126, E128, E129, E130, E131, E136, E138, E139, E140, E141, E146, E148, E149
Diabetes (with complications)	E102, E103, E104, E105, E107, E112, E113, E114, E115, E117, E122, E123, E124, E125, E127, E132, E133, E134, E135, E137, E142, E143, E144, E145, E147
Paraplegia/hemiplegia	G81, G82, G041, G114, G800, G830, G831, G832, G833, G834, G839
Mild liver disease	B18, K73, K74, K700, K701, K702, K703, K709, K717, K713, K714, K715, K760, K762, K763, K764, K768, K769, Z944
Moderate or severe liver disease	K704, K711, K721, K729, K765, K766, K767, I850, I859, I864, I982
Renal disease	N18, N19, N052, N053, N054, N055, N056, N057, N250, I120, I131, N032, N033, N034, N035, N036, N037, Z490, Z491, Z492, Z940, Z992

ICD-10 = International Classification of Diseases, 10th Revision.

Table S2. Indications/reasons for CT.

Indications/reasons	Number of CT scans (total number: 32,650)
Gastric cancer diagnosis and surveillance	31,405
Abdominal pain	115
Colorectal cancer screening	21
Gastrointestinal bleeding	7
Gallbladder or biliary stones	59
Abdominal lymphadenopathy	5
Hematuria	18
Pancreatitis	21
Appendicitis	10
Angiomyolipoma	5
Hepatic benign nodule (cyst, abscess, or hemangioma)	64
Hepatocellular carcinoma surveillance	239
Benign adrenal nodule	28

Renal cyst	33
Inflammatory bowel syndrome	1
Fever of unknown origin	4
Hydronephrosis or urinary stones	54
Abdominal hernia	2
Pancreas cyst	28
Primary biliary cirrhosis	1
Traffic accident	3
Diverticulosis	4
Abdominal aortic aneurysm, abdominal hematoma, ischemic colitis, or venous thrombosis	14
Check-up for malignancy	509

cancers MDPI

Table S3. Risk of intra-abdominal SPMs following abdominopelvic CT scans in selected population subgroups.

Subgroup	≤8 CT scans Incidence rate (per 1,000 person-years)	>8 CT scans Incidence rate (per 1,000 person-years)	HR (95% CI)	*P* for interaction
Age				0.53
<50 years	2.93	14.51	3.69 (0.82–16.56)	
≥50 years	8.18	23.67	2.61 (1.53–4.46)	
Sex				0.06
Male	8.28	20.45	2.03 (1.10–3.76)	
Female	3.39	25.68	7.51 (3.10–18.21)	
Smoking				0.10
Never	5.68	24.77	4.37 (2.35–8.12)	
Past or current smoker	8.71	17.41	1.62 (0.66–3.99)	
CCI				0.16
<2	6.39	22.74	3.25 (1.93–5.49)	
≥2	11.39	17.86	1.47 (0.34–6.24)	
Chronic hepatitis or liver cirrhosis (K74.6ǀK70.3ǀK76.7ǀB18)				0.04
No	6.20	21.33	3.34 (1.91–5.86)	
Yes	21.15	24.68	1.45 (0.48–4.37)	

Estimated from Cox proportional hazard models adjusted for age, sex, smoking status, and Charlson comorbidity index.
SPM, second primary malignancy; HR, hazards ratio; CI, confidence interval; CCI, Charlson comorbidity index; the weighted sum of the following conditions (myocardial infarction, congestive heart failure, peripheral vascular disease, cerebrovascular disease, dementia, chronic pulmonary disease, connective tissue disease, peptic ulcer disease, diabetes without complications, diabetes with complications paraplegia)

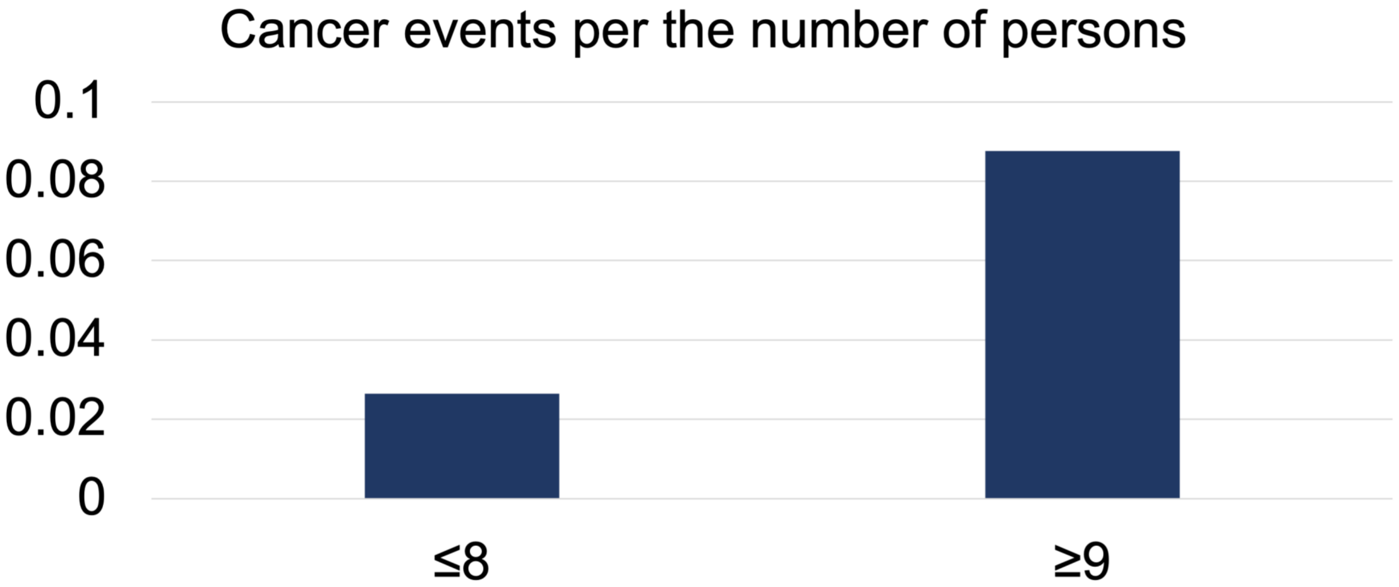

Figure S1. Absolute risks for SPMs according to the number of CT scans.The graph depicts absolute risks (y-axis) between SPMs and the number of abdominopelvic CT scans (x-axis). SPM, second primary malignancy; CT, computed tomography.
